# Esperanza Window Traps for the collection of anthropophilic blackflies (Diptera: Simuliidae) in Uganda and Tanzania

**DOI:** 10.1371/journal.pntd.0005688

**Published:** 2017-06-19

**Authors:** Adam Hendy, Vincent Sluydts, Taylor Tushar, Jacobus De Witte, Patrick Odonga, Denis Loum, Michael Nyaraga, Thomson Lakwo, Jean-Claude Dujardin, Rory Post, Akili Kalinga, Richard Echodu

**Affiliations:** 1 Department of Biomedical Sciences, Institute of Tropical Medicine, Antwerp, Belgium; 2 Evolutionary Ecology Group, Department of Biology, University of Antwerp, Wilrijk, Belgium; 3 Department of Disease Control, London School of Hygiene & Tropical Medicine, London, United Kingdom; 4 Vector Control Division, Ministry of Health, Kampala, Uganda; 5 School of Natural Sciences and Psychology, Liverpool John Moores University, Liverpool, United Kingdom; 6 National Institute for Medical Research, Tukuyu Research Centre, Tukuyu, Tanzania; 7 Faculty of Science, Gulu University, Gulu, Uganda; Washington University School of Medicine, UNITED STATES

## Abstract

There is an increasing need to evaluate the impact of chemotherapeutic and vector-based interventions as onchocerciasis affected countries work towards eliminating the disease. The Esperanza Window Trap (EWT) provides a possible alternative to human landing collections (HLCs) for the collection of anthropophilic blackflies, yet it is not known whether current designs will prove effective for onchocerciasis vectors throughout sub-Saharan Africa. EWTs were deployed for 41 days in northern Uganda and south eastern Tanzania where different *Simulium damnosum* sibling species are responsible for disease transmission. The relative efficacy of EWTs and HLCs was compared, and responses of host-seeking blackflies to odour baits, colours, and yeast-produced CO_2_ were investigated. Blue EWTs baited with CO_2_ and worn socks collected 42.3% (2,393) of the total *S*. *damnosum* s.l. catch in northern Uganda. Numbers were comparable with those collected by HLCs (32.1%, 1,817), and higher than those collected on traps baited with CO_2_ and BG-Lure (25.6%, 1,446), a synthetic human attractant. Traps performed less well for the collection of *S*. *damnosum* s.l. in Tanzania where HLCs (72.5%, 2,432) consistently outperformed both blue (16.8%, 563) and black (10.7%, 360) traps baited with CO_2_ and worn socks. HLCs (72.3%, 361) also outperformed sock-baited (6.4%, 32) and BG-Lure-baited (21.2%, 106) traps for the collection of anthropophilic *Simulium bovis* in northern Uganda. Contrasting blackfly distributions were observed on traps in Uganda and Tanzania, indicating differences in behaviour in each area. The success of EWT collections of *S*. *damnosum* s.l. in northern Uganda was not replicated in Tanzania, or for the collection of anthropophilic *S*. *bovis*. Further research to improve the understanding of behavioural responses of vector sibling species to traps and their attractants should be encouraged.

## Author summary

Using human bait to collect blood-feeding insects is an ethically sensitive issue. Whereas researchers investigating insect-borne diseases such as sleeping sickness, leishmaniasis and malaria have a range of traps at their disposal, those investigating blackflies and river blindness (onchocerciasis) still rely on this method. Alternatives to human bait are needed to monitor disease transmission as onchocerciasis control programmes approach their elimination phase. The recently developed Esperanza Window Trap provides one such possibility. We built these traps based on previously published methods while conducting blackfly research in Uganda and Tanzania in order to evaluate their efficacy and ease of use. Our results show that in Uganda the traps worked well for the collection of *Simulium damnosum*, the blackfly primarily responsible for onchocerciasis transmission in sub-Saharan Africa, but were less effective at collecting the same species in Tanzania. Blackfly behaviour and response to traps will probably vary from one country to another. Esperanza Window Traps show promise for blackfly collections, but further research and development are needed to determine how broadly they can be used.

## Introduction

In 1966, the World Health Organization (WHO) acknowledged a need to develop new sampling techniques to replace human landing collections (HLCs) for the collection of blackfly (Diptera: Simuliidae) species involved in the transmission of *Onchocerca volvulus*, the parasitic filarial nematode responsible for human onchocerciasis [1]. Despite a comprehensive review of adult blackfly collection methods by Service in 1977 [2], subsequent research efforts to meet the needs outlined by the WHO have been limited [3–9]. The primary concern is for the development of a trap to replace HLCs to monitor progress towards onchocerciasis elimination, but an effective trap might also be deployed as a control mechanism in itself to reduce vector populations in support of mass drug administration. The recent development of the Esperanza Window Trap (EWT), used successfully for the collection of host-seeking anthropophilic blackflies in Mexico and Burkina Faso, has provided the possibility of one such viable method [7, 10–13].

### Control and surveillance

Following the implementation of the Mectizan (ivermectin) Donation Program in 1987, methods of onchocerciasis control switched from vector-based interventions to mass drug administration through community directed treatment with ivermectin (CDTI) [14]. Whereas it has been established that ivermectin treatment can eliminate the disease in certain endemic foci, the conditions under which CDTI alone is effective have not been fully explored [15–17]. It is therefore essential that methods for monitoring entomological and parasitological indices of onchocerciasis transmission are available in intervention and post-intervention settings as countries work towards elimination [18, 19]. For EWTs to be effective in evaluating the impact of chemotherapeutic and vector-based programmes, they should collect appropriate numbers of the same vector populations as those biting humans. They should also collect vectors with the same age structure (parity rates) as those biting humans, or collect them in a condition that enables age structures to be calibrated.

The current WHO guidelines for entomological evaluation of *O*. *volvulus* transmission in CDTI settings require that HLCs are used for the collection of anthropophilic blackflies [20, 21]. The method is robust, sensitive, and well accepted by communities, and is therefore preferable to more invasive methods of *O*. *volvulus* surveillance such as Ov-16 serology testing in children [21]. However, human participants collecting biting flies are potentially exposed to a range of vector-borne pathogens, although with appropriate training, the risk is generally considered no higher than for others living in disease endemic areas. Despite this, obtaining the necessary ethical approval can often delay the implementation of research and surveillance programmes [22].

### Available traps

Attempts to develop new, or to utilise or modify existing traps for the collection of host-seeking, anthropophilic blackflies, have been met with mixed or limited success [2]. Light traps [3, 4], sticky traps and silhouettes [23–26], BG-Sentinel traps [7], modified Challier-Lavessiere tsetse traps [5, 6], and other novel traps [27] have been successfully used to collect blackflies in various physiological states, yet repeating collections using these methods has sometimes proved difficult [8, 9].

### Visual attraction

Early investigations into the response of blackflies to long-range visual and olfactory stimuli, including colour, shape, and CO_2_, were mainly confined to Nearctic species including *Simulium venustum* and *Simulium vittatum* [28–32]. Several studies indicate that host-seeking blackflies generally prefer to land on darker colours and matt surfaces [30, 31, 33], and it is also thought low UV reflectance and strong contrast of traps against their background is important in attraction [28, 32, 34]. Comparatively little research has been dedicated to similar investigations for *Simulium damnosum sensu lato* (s.l.), the principal vector of *O*. *volvulus* in Africa. The limited data that exists is consistent with colour-choice experiments for other blackflies, in that host-seeking *S*. *damnosum* s.l. appear to be attracted to dark colours [5, 24, 25, 35]. However, results of behavioural studies should be interpreted cautiously, and Walsh (1980) stresses that they should not be generalised for species other than those being investigated [25, 28]. This is likely to be especially relevant when studying *S*. *damnosum* s.l., a complex of sibling species composed of at least 55 morphologically indistinguishable cytospecies and cytoforms of unknown taxonomic status, each with unique ecological and behavioural traits [36, 37].

### Olfaction

*Simulium damnosum* s.l., like other haematophagous Diptera, are attracted to CO_2_ and host odours [38, 39]. CO_2_ is a powerful mediator of host-seeking behaviour which can greatly enhance blackfly collections [23, 24], yet the biological mechanisms of blackfly attraction to olfactory and visual stimuli are poorly understood [38]. Following experiments in a Cameroonian rainforest, Thompson (1976) demonstrated that the presence of ‘exhaled breath’, industrial CO_2_, and worn clothing, improved trap collections [24, 40]. He concluded that chemicals present in human sweat are likely to be important in attracting *S*. *damnosum* s.l. [40], and that visual and olfactory cues are of greatest importance in attracting savannah and forest sibling species respectively [24]. More recently, EWTs and BG-Sentinel traps baited with worn shirts, trousers (pants) and synthetic chemicals (BG-Lure and octenol) have been shown to be more effective in attracting blackflies than unbaited traps [7]. Young *et al*. (2015) have since used gas chromatography and electroantennography to identify chemicals present in human sweat which are potentially attractive to *S*. *damnosum* s.l. in Burkina Faso and *Simulium ochraceum* s.l. in Mexico [13]. They then demonstrated that EWTs baited with candidate compounds collected 2–3 times the number of these species in the field compared to traps baited with CO_2_ alone, although the authors acknowledge that catch numbers were low and that further research is needed [13].

### Esperanza Window Traps

In 2013, Rodriguez-Pérez *et al*. published results of the development and trial of the EWT in Mexico, which involved investigating the attractiveness of coloured fabrics, CO_2_ sources, and host odours to *S*. *ochraceum* [7]. EWTs constructed using blue fabric outperformed those made with red, yellow and black fabrics when baited with either industrial CO_2_ released at 150-200mL/min, or CO_2_ produced by mixing sugar, yeast (*Saccharomyces cerevisiae*) and water (quantities not specified). There was no statistically significant difference in the number of blackflies collected on traps regardless of the CO_2_ source. With the addition of host odours in the form of a worn shirt or BG-Lure, CO_2_-baited blue EWTs approached the attractiveness of HLCs in one of two trials. In the second trial, the baited EWT was only half as effective as the HLC [7].

Toé *et al*. (2014) further developed the EWT in Burkina Faso for the collection of *Simulium damnosum sensu stricto* (s.str.) and *Simulium sirbanum*, but used black traps baited with BG-Lure and yeast-produced CO_2_ as the basic design [11]. EWTs of differing heights were first compared. ‘Short’ traps, standing within 15cm of the ground were more effective than ‘tall’ traps, although the difference was only statistically significant at one of two sites investigated. The addition of a vertical blue stripe to the black background further enhanced collections, but again, this was only statistically significant at one of the two sites. Short, striped EWTs baited with CO_2_ and BG-Lure caught similar numbers of *S*. *damnosum* s.l. as those baited with CO_2_ and worn trousers. In a final experiment, EWTs baited with CO_2_ and worn trousers collected numbers comparable with HLCs, whereas those baited with worn trousers alone collected numbers similar to unbaited traps. The authors also reported the collection of *Simulium adersi* and *Simulium schoutedeni* from the traps, and questioned the importance of fermentation products other than carbon dioxide in the attraction of vector flies [11].

### Rationale and objectives

The various sibling species of the *S*. *damnosum* complex are behaviourally and ecologically unique in traits such as breeding habitats, dispersal capabilities, degree of anthropophily, and their capacity to transmit disease [37]. It is not yet known whether different sibling species will respond differently to EWTs, and whether current trap designs will prove to be effective for *S*. *damnosum* s.l. collections throughout onchocerciasis affected areas of sub-Saharan Africa. This study therefore aimed to compare the relative efficacy of EWTs with HLCs for the collection of anthropophilic blackflies in onchocerciasis transmission zones of Uganda and Tanzania, where different sibling species of the *S*. *damnosum* complex are responsible for disease transmission. Responses of host-seeking blackflies to odour baits, colour schemes, and yeast-produced CO_2_ were also investigated.

## Materials and methods

### Study area

Experimental work took place for a total of 41 days at five locations in Uganda (26 days), and one in Tanzania (15 days), between 28 June 2015 and 19 September 2016 (Table 1).

**Table 1:** Blackfly collection locations and distance from nearest known breeding sites. Alt. = Altitude, Dist. = Distance.

Country	District	Location	Coordinates		Alt.	Date	Nearest Known Breeding Sites	Dist.
Uganda	Lamwo	Apyeta Bridge	N 03°18.005’	E 032°21.705’	691m	Jul 2015	Achwa River	0km
		Beyogoya	N 03°17.648’	E 032°29.708’	845m	Jul 2015	Achwa River	7.5km
	Moyo	Gwere Luzira	N 03°39.827’	E 031°48.056’	980m	Jul 2015	Nile (S. Sudan)	16km
		Pamulu	N 03°40.758’	E 031°49.452’	1066m	Jul 2015	Nile (S. Sudan)	13km
	Nwoya	Ayago Bridge	N 02°25.907’	E 032°0.452’	897m	Jun 2015	Ayago River	11km
			N 02°25.907’	E 032°0.452’	897m	Aug 2015	Ayago River	11km
			N 02°25.974’	E 032°0.454’	898m	Sep 2016	Ayago River	11km
Tanzania	Ulanga	Chikuti	S 08°36.175’	E 036°44.072’	459m	Jun 2016	Mbalu River	5km

Collections were made in the districts of Lamwo, Moyo and Nwoya in the Madi/Mid-North onchocerciasis transmission zones of northern Uganda. Savannah grassland predominates and *S*. *damnosum* s.str. is thought to be the principal vector of *O*. *volvulus* [41, 42]. Small numbers of *S*. *sirbanum* also breed along the Pager River northeast of Kitgum [43]. In addition, a member of the *Simulium bovis* species-group also forms a significant proportion of the anthropophilic blackfly population in the Mid-North [44]. Both *S*. *damnosum* s.l. and *S*. *bovis* occupy similar breeding habitats [45, 46]. In Lamwo district, these are mainly along the larger rivers including the Achwa (Aswa) and Pager [47, 48]. In Moyo, there is thought to be little local breeding of *S*. *damnosum* s.l., and it is likely that biting blackflies migrate from a series of rapids along the Nile in neighbouring South Sudan [43, 49]. The Murchison Nile forms the southern boundary of Nwoya district and is a major source of blackfly breeding [49]. There are historical reports of *S*. *damnosum* s.l. breeding along the Ayago River, a tributary of the Nile, and the Kibaa and Murchison River tributaries have also been cited as possible sources of infestation [49, 50]. Rainfall lasts from April to November, with peaks occurring early and late in the rainy season. The climate is hot and dry from December to March [51].

Collections in Tanzania were made at Chikuti on the north side of the Mahenge Mountains in the Mahenge onchocerciasis transmission zone of Ulanga district. The area is characterised by Precambrian limestone, and the presence of riverine, dry lowland and submontane forests [52]. The mountains are drained by numerous stony streams and rivers that are favourable to blackfly breeding [53]. Again, the principal vector of onchocerciasis is *S*. *damnosum* s.l. [35]. The cytoforms present in Mahenge are ‘Nkusi’, *Simulium plumbeum* (= ‘Hammerkopi’ and ‘Ketaketa’), ‘Sebwe’ and ‘Turiani’ [35, 54, 55]. ‘Nkusi’ is thought to be the predominant anthropophilic species, and *S*. *plumbeum* may have a limited role in human biting. Both ‘Sebwe’ and ‘Turiani’ are zoophilic [35, 54]. *Simulium nyasalandicum* (originally reported as *S*. *woodi*) also contributes to biting in small numbers, mainly in the south of the transmission zone [35, 56]. Rainfall lasts from November to May, and peaks between March and May. The dry season lasts from June to October [35, 52].

### Basic trap design

**Fig 1:**
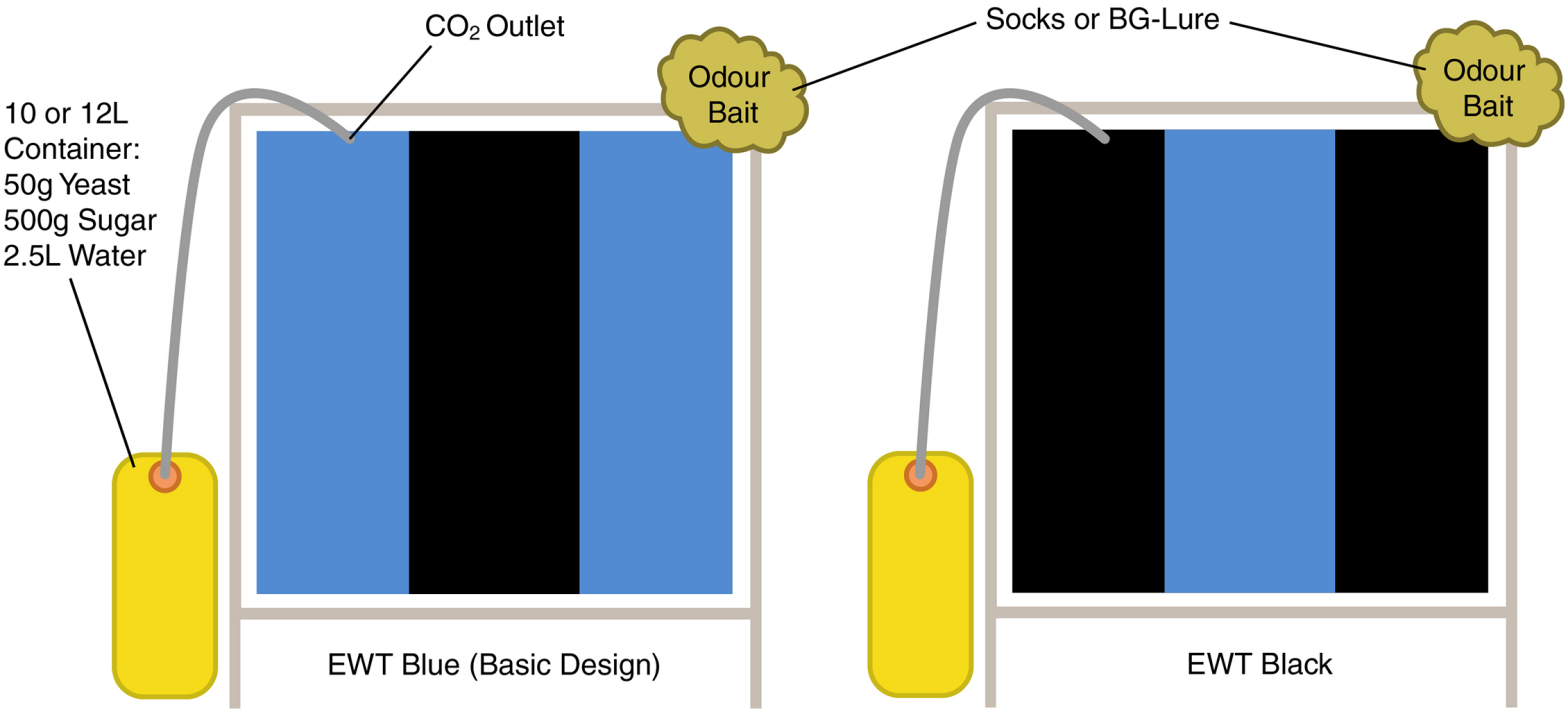
Blue and black trap designs showing position of CO_2_ and odour baits. Blue screens with a black vertical stripe (basic design) were used for all trapping experiments in Uganda. Black screens with a blue vertical stripe were additionally used in Tanzania.

Traps were constructed using locally-sourced materials. Frames were composed of a light-gauge steel and trap faces measured approximately 1m^2^ (Fig 1). Traps stood on 0.25m sharpened legs which were easily pushed into the ground. The basic design included a blue tarpaulin screen that was hung tightly inside the frame. Blue was chosen as the base-colour as blue traps yielded the greatest number of blackflies during collections by Rodriguez-Pérez *et al*. (2013) in Mexico [7]. A black central stripe ⅓ the width of the blue screen was painted onto the trap using a matt black emulsion (Sadolin Paints (U) Limited, Uganda) during initial experiments in Uganda in 2015. The paint was allowed to dry for two days before traps were deployed. During subsequent collections in Tanzania and Uganda (2016), the black paint was replaced with black tarpaulin which was sewn together with the blue tarpaulin to form the screen. A CO_2_ outlet and host odour attractants were attached to the top corners of the EWT frame (Fig 1). Traps were covered with a black plastic sheet when not in use.

### Adhesives

Tangle-Trap insect trap coating paste (Contech, Victoria, BC, Canada) was used to coat EWTs in Uganda. It was not possible to acquire the same product for trapping work in Tanzania due to manufacturing problems. EWTs in Tanzania were therefore coated with Temmen-Insektenleim (Temmen GmbH, Hattersheim, Germany). Both products were thinned using ≈150mL locally purchased white spirit (Sadolin Paints (U) Limited, Uganda), before being applied to traps at least 24h prior to their deployment.

### CO_2_ production

A sugar-yeast based source of carbon dioxide was produced in the field following methods outlined by Smallegange *et al*. (2010) [57]. However, quantities of ingredients were adjusted to provide sufficient CO_2_ output (>80mL/min for at least 11 hours) following incubation at 30°C during preliminary laboratory experiments (S1 Fig). Dry baker’s yeast (50g), sugar (500g) and water (2.5L) were mixed in 10L (Uganda) or 12L (Tanzania) containers immediately prior to blackfly collections commencing. PVC tubing extended from a hole in the container to an outlet at a top corner of the EWT. Containers were briefly shaken before being placed next to traps. Fresh sugar-yeast mixtures were prepared each day by community members assisting with blackfly collections.

### Host odour attractants

Traps were either baited with host odours emanating from a pair of worn socks, or BG-Lure (Biogents AG, Regensburg, Germany), a synthetic mosquito attractant containing chemicals found on human skin (ammonia, lactic acid, and caproic acid) [58]. Worn socks were provided by villagers in exchange for a new pair of socks, and were tied to the top corner of the EWT opposite the CO_2_ outlet and replaced every three days. Worn socks have been shown to be effective for up to 8 days for the collection of mosquitoes [59].

### Human landing collections

HLCs were made by trained community-based participants following standard methods [20]. A team of two people worked alternate hours between 07:00 and 18:00, collecting blackflies landing on their exposed legs. Flies were collected in individual tubes and hourly catches were recorded.

### Specimen preservation and identification

Blackflies were removed from EWTs using forceps after applying a drop of white spirit to specimens in order to partially dissolve the adhesive. A 10x magnification hand lens was used to verify identification of insects where necessary. All blackflies were preserved in >95% ethanol and were identified in the laboratory using morphological keys in Freeman & De Meillon (1953) [60]. The member of the *S*. *bovis* species-group present in northern Uganda was identified based on the morphology of male pupae collected at Apyeta Bridge in 2015. To confirm identification, specimens were compared with reference material at the Natural History Museum, London, UK. The identity of adult *S*. *bovis* group flies collected on traps and by HLC was inferred based on the pupal identifications. Biting flies other than blackflies were removed from traps and preserved during collections made in 2016 only.

### Study design

#### Odour baits

Blackfly collections were made for 21 days at five locations in Lamwo, Moyo and Nwoya districts of northern Uganda between June and August 2015, to compare the efficacy of EWTs (basic design) baited using CO_2_ and either worn socks or BG-Lure, with HLCs. At each location, precise vector collection sites were identified with the assistance of community members according to where blackfly biting was already known. A day was spent training participants in HLC methods and also to prepare CO_2_ mixtures for baiting traps. Three collection sites were selected at each location for the deployment of 1) a team of two people to make HLCs, 2) two EWTs baited with CO_2_ and BG-Lure (EWT BG-Lure), and 3) two EWTs baited with CO_2_ and worn socks (EWT Socks). EWTs were placed in pairs, at right-angles to one another, in an attempt to maximise their visibility. HLC and EWT collections were made simultaneously between 07:00 and 18:00 for a minimum of three days (or in multiples of three days) at each location. Collection sites were at least 30m apart and HLCs and EWTs were rotated daily in a 3x3 randomised Latin square design in order to minimise interference and collection site bias respectively. Blackflies were removed from EWTs each day at approximately 11:00, 14:00 and 17:00 to minimise the impact of desiccation on specimen quality. Daily blackfly catches were compared for each method.

#### Colour schemes

Blackfly collections were made for 15 days at a single location near Chikuti village on the northern side of the Mahenge Mountains in Tanzania in June 2016, to compare the efficacy of EWTs of different colour schemes, with HLCs. Three collection sites were selected in a cultivated field approximately 0.5km from the village centre. Collection methods included 1) a team of two people to make HLCs, 2) two blue EWTs with a black central stripe (EWT Blue), and 3) two black EWTs with a blue central stripe (EWT Black). The EWT Black was similar to the design previously used by Toé *et al*. (2014) in Burkina Faso [11]. Each EWT was baited with CO_2_ and worn socks as previously described. Again, EWTs were placed in pairs, at right-angles to one another. HLC and EWT collections were made simultaneously between 07:00 and 18:00 each day and blackflies were removed from EWTs at approximately 10:00 and 17:00. Collection sites were at least 50m apart and HLCs and EWTs were rotated daily in a 3x3 randomised Latin square design. Daily blackfly catches were compared for each method.

#### Yeast-produced CO_2_

Blackfly collections were made for 5 days at Ayago Bridge in Uganda in September 2016, to compare the efficacy of EWTs (basic design) baited with either a freshly prepared sugar-yeast mixture (EWT CO_2_+), or a mixture that had been prepared 5 days in advance and was no longer producing CO_2_ (EWT CO_2_-). No other odour baits were used in this experiment. Provisional laboratory observations demonstrated that CO_2_ production was <80mL/min after exposing sugar-yeast mixtures to continuous temperatures of 25°C, 30°C and 35°C for 12h (S1 Fig). The amount of gas produced after 5 days would therefore be negligible. Two collection sites were prepared approximately 50m apart by clearing vegetation adjacent to the Ayago River. One trap was placed at each site and collections were made between 07:00 and 18:00 each day. Blackflies were removed at approximately 11:00, 14:00 and 17:00 each day and traps were rotated daily as in previous experiments. Daily blackfly catches were compared for each method.

#### Blackfly distribution

In response to observations that *S*. *damnosum* s.l. were attracted to the lower parts of EWTs during odour bait experiments in Uganda in 2015, attempts were made to quantify blackfly distribution on traps during subsequent colour and CO_2_ experiments in Uganda and Tanzania in 2016. Small holes were made in EWT screens to divide the surface into nine approximately equal squares. The number of blackflies removed daily from each square was recorded for each trap type. Counts from corresponding squares on each side of the trap were combined. Blackflies were preserved daily according to trap type, rather than for each square. Reported blackfly counts on each square are therefore for all blackfly species and not individual species.

### Statistical analysis

In all experiments, blackfly count was the response variable and was modelled as a function of trap type, the main covariate of interest. Location, collection site and rainfall were included as additional covariates. A generalized linear framework with a negative binomial distribution was used to take into account the overdispersion observed in the count data. The Akaike Information Criterion was used to select the most appropriate model for each data set, and models were verified by means of diagnostic plots. When more than one anthropophilic blackfly species was active at a study location, data for each species were analysed separately. Data were excluded from analysis for a particular species if blackfly collections were low (<5/day using all methods), or if the species was absent. The negative binomial model was also used to analyse the distribution of blackflies on traps, and to investigate interactions between blackfly attachment on columns and rows. Heat maps of blackfly attachment to traps were produced using log transformed data to improve graphical representation of blackfly distribution. Analyses were performed within the R version 3.3.2 statistical computing environment [61].

### Ethics statement

Blackfly collections involving human participants were subject to review and approval by the institutional review board at the Institute of Tropical Medicine, Antwerp, Belgium (960/14, 1089/16); the Higher Degrees, Research and Ethics Committee, Makerere University School of Public Health, Kampala, Uganda (2014/244); and the Medical Research Coordinating Committee at the National Institute for Medical Research, Dar es Salaam, Tanzania (NIMR/HQ/R.8a/Vol.IX/2212). Formal approval to conduct studies in Uganda was granted by the Uganda National Council for Science and Technology (HS 1701). All participants were adults over the age of 18 years who provided written informed consent.

## Results

A total of 13,152 female blackflies (*Simulium* spp.) were collected during the study using all methods (Table 2). Of these, 10,652 were preserved and identified. The remaining 2,500 were discarded when catch numbers were either too high to remove and preserve all specimens, or the species composition was known to be >99% *S*. *damnosum* s.l. based on previous collections. No male blackflies were caught by HLCs or EWTs during the study. In 2015, *S*. *damnosum* s.l. comprised >99.9% (5,656/5,663) of all blackflies collected in Moyo and Nwoya districts of northern Uganda, but only 1.4% (7/506) of those collected in Lamwo district. The remaining 98.6% (499/506) were identified as *S*. *bovis sensu* De Meillon (1930) [60]. In 2016, a further 3,476 blackflies were collected on EWTs in Nwoya district, but only 1,201 were preserved. Of these, 99.6% (1,196/1,201) were identified as *S*. *damnosum* s.l. and it was presumed that a similar proportion of the 2,275 non-preserved flies were the same species. *Simulium damnosum* s.l. comprised 96.3% (3,161/3,282) of all blackflies preserved and identified from collections made in Tanzania using all methods. Other Simuliidae present in Tanzania included *S*. *vorax*, *S*. *adersi*, *S*. *hirsutum* and a number of small unidentified species.

**Table 2:** Summary data showing number of blackflies of each species collected using all methods.

Year	Country	District	Location	Trap Days	Total Blackflies	Total Preserved	*damnosum*	*bovis*	*vorax*	*adersi*	*hirsutum*	Other	Not Preserved
2015	Uganda	Lamwo	Apyeta Bridge	3	327	327	1	326	0	0	0	0	0
			Beyogoya	3	179	179	6	173	0	0	0	0	0
		Moyo	Gwere Luzira	3	766	766	766	0	0	0	0	0	0
			Pamulu	3	935	935	929	0	0	0	0	6	0
		Nwoya	Ayago Bridge	9	3962	3962	3961	0	0	0	0	1	0
2016	Uganda	Nwoya	Ayago Bridge	5	3476	1201	1196	0	0	0	0	5	2275^b^
2016	Tanzania	Ulanga	Chikuti	15	3507	3282	3161	0	8	11	5	97	225^C^
			**Total**	**41**	**13152**	**10652**	**10020**	**499**	**8**	**11**	**5**	**109**^a^	**2500**

^a^Small blackflies unidentifiable morphologically using Freeman & De Meillon (1953).

^b^Specimens presumed to be *S*. *damnosum* s.l. based on known species composition at Ayago Bridge.

^c^Specimens removed from EWT Blue without being preserved on a single collection day when catch numbers were unexpectedly high. Based on the frequency distribution of the observed specimens it was estimated that 194 of the 225 specimens were *S*. *damnosum* complex.

### Odour baits

**Fig 2:**
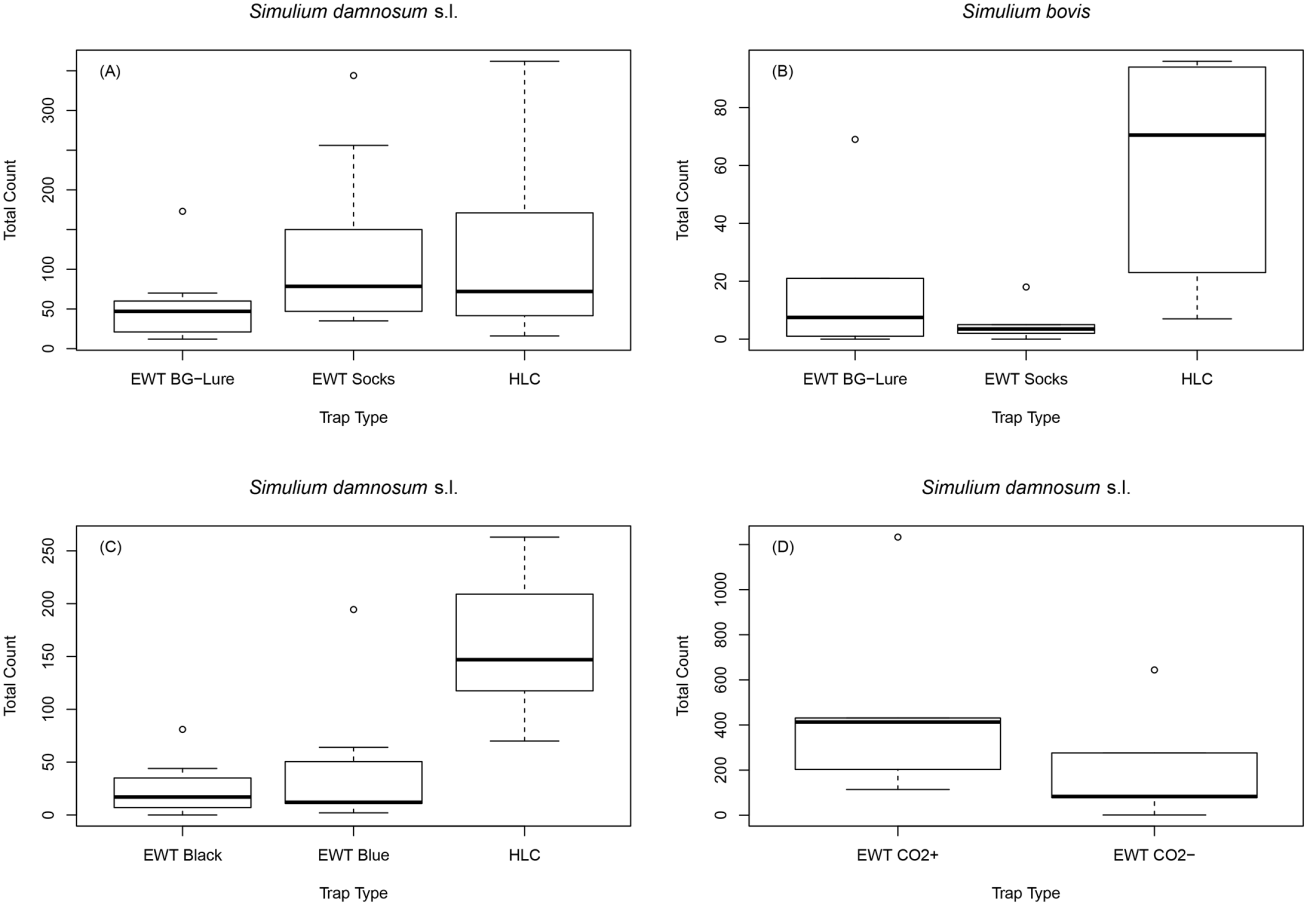
Median values and interquartile ranges of daily *S*. *damnosum* s.l. and *S*. *bovis* collections made using EWTs and HLCs. (A) *S*. *damnosum* s.l. collections made using BG-Lure and sock-baited EWTs in northern Uganda, 2015 (B) *S*. *bovis* collections made using BG-Lure and sock-baited EWTs in northern Uganda, 2015 (C) *S*. *damnosum* s.l. collections made using black and blue EWTs in Tanzania, 2016 (D) *S*. *damnosum* s.l. collections made using fresh (CO_2_+) and pre-prepared (CO_2_-) sugar-yeast sources of CO_2_ in northern Uganda, 2016.

Pairs of traps baited with CO_2_ and worn socks (EWT Socks) were as effective as the HLC for the collection of *S*. *damnosum* s.l. in northern Uganda, while pairs of traps baited with CO_2_ and BG-Lure (EWT BG-Lure) were the least effective overall (Fig 2A). However, there was a significant interaction effect of trap type and location on blackfly collections (p = 0.002). The EWT Socks outperformed the HLC and EWT BG-Lure at Ayago Bridge and Gwere Luzira, whereas the reverse was true at Pamulu. After 15 trap days, the EWT BG-Lure collected 25.6% (1,446), the EWT Socks 42.3% (2,393), and the HLC 32.1% (1,817) of the total *S*. *damnosum* s.l. catch (Table 3).

**Table 3:** Summary data of *S*. *damnosum* s.l. and *S*. *bovis* collections for each trap type.

Year	Country	Species	Trap Days	Trap Type	Median	IQR	Min.	Max.	Total	% Total
2015	Uganda	*S*. *damnosum* s.l.	15	EWT BG-Lure	47	39	12	173	1446	25.6
				EWT Socks	78.5	97.5	35	344	2393	42.3
				HLC	72.0	129.5	16	362	1817	32.1
2015	Uganda	*S*. *bovis*	6	EWT BG-Lure	7.5	20	0	69	106	21.2
				EWT Socks	3.5	3	0	18	32	6.4
				HLC	70.5	71	7	96	361	72.3
2016	Uganda	*S*. *damnosum* s.l.	5	EWT CO_2_+	413	228	114	1233	2394	68.9
				EWT CO_2_-	83	198	1	644	1082	31.1
2016	Tanzania	*S*. *damnosum* s.l.	15	EWT Black	20	32	5	95	360	10.7
				EWT Blue	19	42	2	194	563	16.8
				HLC	147	91.5	70	263	2432	72.5

There was a significant effect of trap type on the number of *S*. *bovis* collected in Lamwo district (p = 0.008). Unlike for the collection of *S*. *damnosum* s.l., there was no interaction effect of trap type and location on collections (p = 0.58) (Fig 2B). The HLC clearly outperformed EWTs of both types at Apyeta Bridge and Beyogoya (p<0.001), and there was weak evidence to suggest the EWT Socks was the least effective trap overall (p = 0.074). After 6 trap days, the EWT BG-Lure collected 21.2% (106), the EWT Socks 6.4% (32), and the HLC 72.3% (361) of the total *S*. *bovis* catch (Table 3).

### Colour schemes

More than 99% of blackflies recovered from EWTs in Uganda were morphologically indistinguishable from those collected by HLC. This was not the case in Tanzania where *S*. *damnosum* s.l. comprised 100% of the catch by HLC, but only 86.3% (360/417) and 85.6% (563/658) of the catch on the EWT Black and EWT Blue traps respectively. There was a significant effect of trap type on *S*. *damnosum* s.l. collections at Chikuti (p<0.001) where the HLC clearly and consistently outperformed EWTs of each colour scheme (Fig 2C). There was no overall difference in efficacy between the EWTs, and despite the EWT Blue outperforming the EWT Black at two of the three collection sites, there was insufficient evidence to suggest *S*. *damnosum* s.l. preferred one colour scheme over another (p = 0.28). After 15 trap days, the EWT Black collected 10.7% (360), the EWT Blue 16.8% (563), and the HLC 72.5% (2,432) of the total *S*. *damnosum* s.l. catch (Table 3).

### Yeast-produced CO_2_

Rainfall restricted trapping to five days at Ayago Bridge in Uganda during September 2016, although this was sufficient to demonstrate that freshly prepared sugar-yeast mixtures (producing CO_2_) enhanced *S*. *damnosum* s.l. collections (p<0.001) (Fig 2D). After 5 trap days, the EWT CO_2_+ collected 68.9% (2,394) and the EWT CO_2_- 31.1% (1,082) of the total *S*. *damnosum* s.l. catch (Table 3). Trap site was a significant explanatory variable (p<0.001) and blackfly activity was noticeably higher at one of the two collection sites. Both sites were situated in areas of cleared bush surrounded by tall vegetation, although the most productive site had greater exposure to direct sunlight. When exposed to direct sunlight, *S*. *damnosum* s.l. would primarily land on the shaded side of traps.

### Blackfly distribution

The vertical distribution of blackflies (all species) was similar for both the EWT CO_2_+ and EWT CO_2_- in Uganda where 62.8% and 66.9% of specimens were removed from the bottom rows of respective traps (Table 4). Blackfly numbers decreased with increasing height on the traps (p<0.001) regardless of whether CO_2_ was present or absent.

**Table 4:** Summary data showing blackfly distribution on rows and columns of traps, including mean daily catch and standard errors (SE).

Country	Trap Days	Trap Type	Row	Mean Daily Catch^a^ (SE)	% Total	Column	Mean Daily Catch^a^ (SE)	% Total
Uganda	5	EWT CO_2_+	Top	60.8 (24.2)	12.7	Left	227.4 (105.3)	47.5
			Middle	117.4 (49.8)	24.5	Middle	171.8 (65.2)	35.9
			Bottom	300.6 (124.5)	62.8	Right	79.6 (29.4)	16.6
	5	EWT CO_2_-	Top	15.8 (7.1)	7.3	Left	53 (19.4)	24.5
			Middle	55.8 (27.7)	25.8	Middle	88 (49.3)	40.7
			Bottom	144.8 (82.7)	66.9	Right	75.4 (49.2)	34.8
Tanzania	12	EWT Blue	Top	31.7 (14.9)	60.4	Left	25.3 (9.6)	48.2
			Middle	11.4 (3.8)	21.8	Middle	7.8 (2.0)	14.9
			Bottom	9.3 (2.8)	17.8	Right	19.3 (8.1)	36.9
	12	EWT Black	Top	18.7 (6.1)	58.0	Left	11.9 (2.7)	37.0
			Middle	7.8 (1.4)	24.1	Middle	10.1 (2.3)	31.3
			Bottom	5.8 (0.9)	17.9	Right	10.2 (3.5)	31.6

^a^All blackfly species.

**Fig 3:**
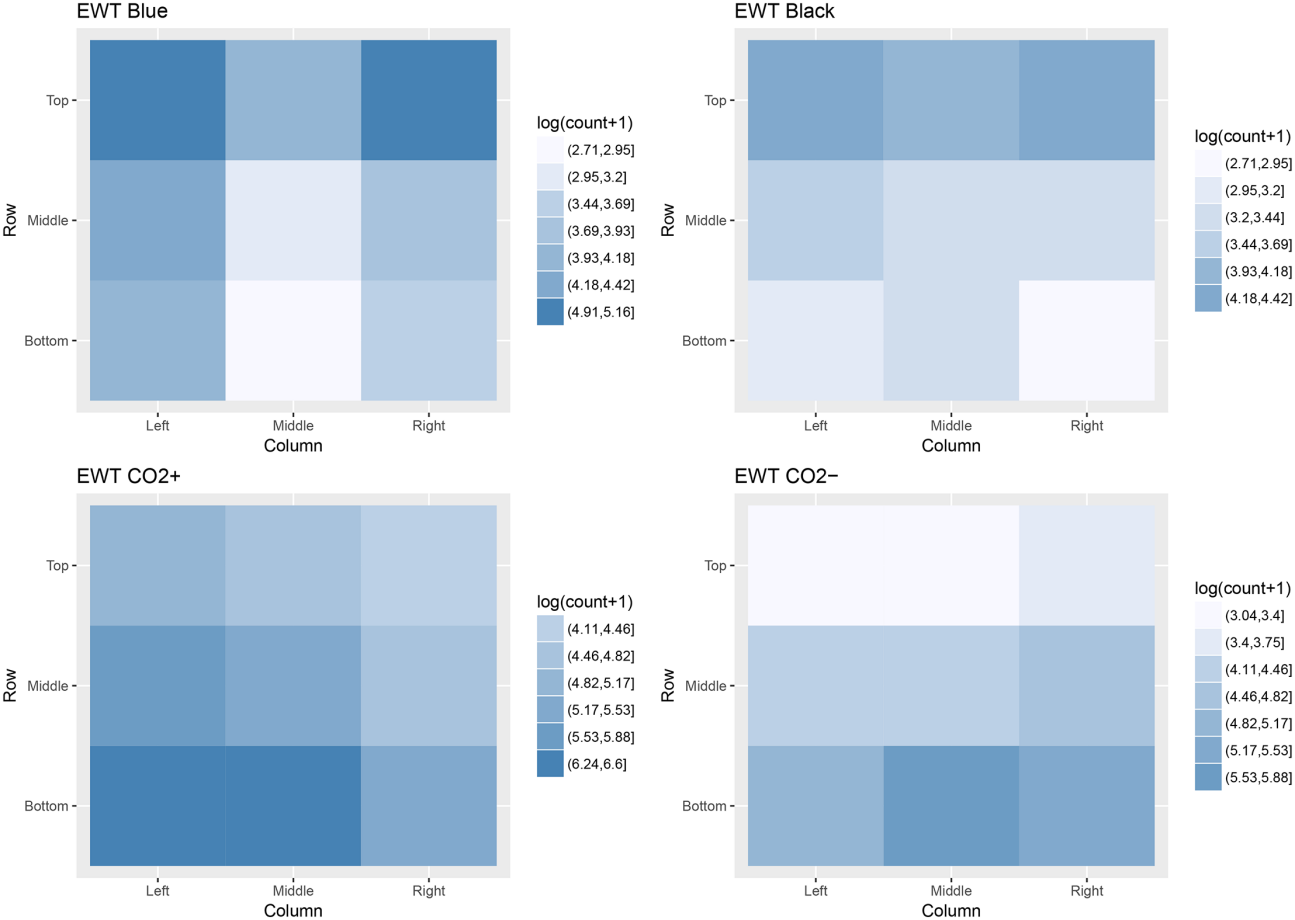
Heat maps illustrating distribution of all blackfly specimens collected on EWTs in Tanzania (EWT Blue and EWT Black) and Uganda (EWT CO_2_+ and EWT CO_2_-) in 2016.

In contrast, blackflies (all species) in Tanzania showed greater attraction to the top row of EWTs (p<0.001) (Table 4). Again, the percentage of blackflies differed little between the traps, with 60.4% and 58.0% being removed from the top rows of the EWT Blue and EWT Black respectively. Blackfly numbers decreased with decreasing height on EWTs of both colour schemes (p = 0.021). The horizontal distribution of blackflies on the EWT Blue indicated a preference towards the outer columns where the CO_2_ outlet (left) and worn socks (right) were located (p = 0.002). There was also a slight preference towards the left column on the EWT Black, although blackflies were otherwise more evenly distributed across columns than on the EWT Blue. Log transformed counts of blackfly distribution are illustrated in Fig 3.

### Other biting flies

Only five biting flies other than blackflies were removed from traps in Tanzania and all were Tabanidae of the genera *Haematopota* and *Tabanus*. Biting flies were more diverse and abundant at Ayago Bridge in Uganda and included both male and female *Glossina f*. *fuscipes* and *Glossina pallidipes*. Glossinidae were identified to species using morphological and molecular methods in the laboratory of Prof Stephen Torr (Liverpool School of Tropical Medicine, UK). *Stomoxys calcitrans* and several unidentified *Haematopota* and *Tabanus* species were also collected (Table 5). The biting flies recovered from traps were of sexes exhibiting anthropophilic behaviour for each species.

**Table 5:** Biting flies other than blackflies removed from traps in Tanzania and Uganda, 2016.

Date of Collection	Country	Location	Family	Genus	Species	Sex	Number
June 2016	Tanzania	Chikuti	Tabanidae	*Haematopota*	sp.	♀	1
				*Tabanus*	sp.	♀	4
September 2016	Uganda	Ayago Bridge	Glossinidae	*Glossina*	*f*. *fuscipes*	♀	9
						♂	14
					*pallidipes*	♀	3
						♂	10
			Muscidae	*Stomoxys*	*calcitrans*	*♀*	4
			Tabanidae	*Haematopota*	sp.	♀	7
				*Tabanus*	spp.	♀	2

## Discussion

It was initially stated that for EWTs to be viable for *O*. *volvulus* surveillance, they should collect appropriate numbers of the same vector populations as those biting humans.

### Odour baits

Whereas pairs of blue EWTs baited with CO_2_ and BG-Lure appeared to be less effective than in previous studies in Mexico and Burkina Faso [7, 11], those baited with CO_2_ and worn socks regularly collected numbers comparable with HLCs in northern Uganda. A notable exception was at Pamulu, where the EWT Socks caught the fewest flies. Blackfly activity varied greatly from site to site at each location, and it rained on the day the EWT Socks was positioned at the site with highest activity at Pamulu. The negative impact of rain on trap performance was compounded by the limited number of catching days (3) at this location. There was no rain at Gwere Luzira, so traps were unaffected. In addition, the higher number of trapping days (9) at Ayago Bridge meant the impact of rain on overall trap performance was less apparent than at Pamulu.

In contrast to the success of the Ugandan collections, EWTs baited with CO_2_ and worn socks performed relatively poorly compared to HLCs for the collection of *S*. *damnosum* s.l. in Tanzania. It is not clear why, although given that different *S*. *damnosum* sibling species were present in the study areas of each country, it seems plausible that they might respond differently to traps. The host-oriented behaviour of Glossinidae has been extensively studied and there is evidence of both interspecific and intraspecific variation in response to host kairomones [62, 63]. Similar differences in behavioural response may exist for the many sibling species of the *S*. *damnosum* complex, and the recent study of blackfly attraction to human semiochemicals by Young *et al*. (2015) should provide a good starting point for further research [13]. In the meantime, the most appropriate odour bait is probably worn clothing, that is easy to obtain and reflects odour profiles of local populations.

EWTs performed poorly for the collection of *S*. *bovis* in northern Uganda. This is a species that generally feeds on cattle, although frequent human biting has been reported in the past from Nigeria and northern Cameroon [45, 64]. It has been proposed that anthropophily may develop in the absence of its usual bovine host [45]. Pairs of EWTs baited with worn socks collected just 6.4% (32/499) of the total *S*. *bovis* catch (Table 3). EWTs baited with BG-Lure performed slightly better, collecting 21.2% (106/499) of the total catch. However, the difference in trap efficacy can probably be explained by the presence of a herd of cattle, rather than attraction to the lures. Of the 106 *S*. *bovis* collected over six days on traps baited with BG-Lure, 65.1% (69) were collected on a single day at Apyeta Bridge. On that day, cattle passed within a few metres of the BG-Lure-baited traps. The observed number of blackflies was noticeably higher on these traps immediately after the cattle had passed. Whereas flies “carried” by the cattle might have dispersed and enhanced collections on all trap types, the impact was much more evident on those closest to the herd. A similar event occurred at Gwere Luzira where the presence of cattle also coincided with a high (240) *S*. *damnosum* s.l. catch on sock-baited EWTs. Again, there were noticeable differences in the number of blackflies on these traps before and after the event. Such confounding factors will need to be taken into consideration if attempting to calibrate trap collections with human biting rates. Care will also need to be taken to place traps away from shared animal hosts of human biting blackflies.

Uniformity of experiments would have been improved by standardising the washed status of HLC participants and also the amount of time socks were worn for in advance of trapping. Baiting traps with socks from both HLC participants might also have reduced bias caused by variation in human attractiveness to blackflies [59].

### Colour schemes

HLCs consistently outperformed EWTs of each colour scheme in Tanzania. Possible reasons for differences in trap-efficacy observed between countries are discussed in the following sections. As a result of the poor relative performance of traps in Tanzania, there was insufficient evidence to demonstrate that *S*. *damnosum* s.l. preferred one colour scheme over the other. Further investigations of colour preference among *S*. *damnosum* sibling species are warranted.

### Yeast-produced CO_2_

Freshly prepared sugar-yeast mixtures clearly enhanced the number of blackflies collected on EWTs. Despite concerns raised that fermentation products other than CO_2_ are likely to attract vector flies other than those seeking a blood meal, the impact appears to have been negligible [11, 57]. Since no male blackflies were collected on traps, despite non-vector species breeding in the adjacent river, it is likely that CO_2_ is the most important compound in attraction. However, it should be noted that various Hymenoptera and Diptera were frequently attracted to the jerry can containing the sugar-yeast mixture. Comparing the parity rates and gonotrophic status of HLC and EWT-collected flies would help further clarify whether sugar-yeast mixtures are only attracting host-seeking vectors.

### Blackfly distribution

The contrasting distribution of blackflies of all species on EWTs in Uganda and Tanzania appears to indicate differences in *S*. *damnosum* s.l. behavioural response, although differences in species composition present obvious limitations to the study.

Perhaps the simplest explanation would be to refer to the previously mentioned work of Thompson (1976) in Cameroon [24]. If savannah sibling species are more reliant on visual host-seeking cues [24], are naturally inclined to fly close to the ground [38, 65, 66], and tend to land low on their host [65, 66], this could sufficiently explain the distribution of blackflies on traps in Uganda. The percentage of blackflies removed from the bottom (62.8%/66.9%) and middle (24.5%/25.8%) rows of the EWT CO_2_+ and EWT CO_2_- (Table 4), compares well with a study of savannah *S*. *damnosum* s.l. in northern Cameroon [66]. Here, Renz and Wenk (1983) demonstrated that most flies fed on the ankles (53%/51%) and calves (28%/27%) of standing and sitting volunteers respectively [66]. The percentage of blackflies removed from the top (60.4%/58.0%) and middle (21.8%/24.1%) rows of the EWT Blue and EWT Black at Chikuti in Tanzania shows a considerably contrasting distribution. It could be that the behaviour of sibling species present in the Mahenge Mountains more closely resembles the forest sibling species described by Thompson (1976) [24]. It is possible that they are more reliant upon olfactory cues when host-seeking, explaining why greater numbers were removed from the top rows of traps where odour baits were positioned [24].

Host preferences of sibling species present in Mahenge may offer another explanation. It is known that the vertical distribution of haematophagous Diptera can be influenced by their hosts [67, 68]; that no blackfly species is exclusively anthropophilic [37], and that degrees of anthropophily vary among human biting members of the *S*. *damnosum* complex [69]. Little is known about the respective blood hosts of *S*. *damnosum* s.l. in Mahenge, although ‘Nkusi’ is probably responsible for the majority of human biting [35]. It is also known to feed on cattle in addition to humans in western Uganda [70]. The remaining cytoforms, *S*. *plumbeum*, ‘Sebwe’ and ‘Turiani’ are either mainly or entirely zoophilic [35, 54], and zoophilic blackflies can also be specific in their preferred feeding sites on a host [71]. For example, East African *S*. *vorax* and *S*. *nyasalandicum* prefer to bite the ears and underside of cattle, respectively [71]. Many ornithophilic blackfly species also prefer to bite the area around the head and neck of their hosts [72, 73]. Studies of Glossinidae have shown that odour-oriented responses attract flies towards their hosts, but final responses are to visual cues [63, 74]. Again, similar mechanisms of host-location might also exist for blackflies [63].

It is not known whether EWTs were sampling the same sibling species as HLCs during studies in Uganda and Tanzania. PCR-based identification of *S*. *damnosum* s.l. collected using each method might have highlighted any differences in sibling species composition [75]. The use of unbaited EWTs, or EWTs with odour baits positioned at different heights, might have clarified the importance of visual and olfactory cues in each study area. Preserving blackflies according to the area of the trap on which they landed, rather than according trap type, would have enabled the distribution of *S*. *damnosum* s.l. and other species to be represented more accurately. Also, blood meal analyses of flies collected on EWTs or breeding in nearby rivers might have yielded information about host preference.

### Absence of males

The lack of male *S*. *damnosum* s.l. and *S*. *bovis* on traps might suggest that EWTs specifically target host-seeking females, but this should be considered in relation to the distance of collection sites from breeding sites. Little is known about dispersal distances of male blackflies, although it is generally thought they disperse shorter distances than females [71, 76]. With the exception of adult collection sites at Apyeta Bridge which were adjacent to the Achwa River, those at Pamulu (13km), Gwere Luzira (16km), Beyogoya (7.5km) and Ayago Bridge (11km), were a considerable distance from places of known *S*. *damnosum* s.l. breeding (Table 1). At Chikuti, they were also 5km from known breeding sites in the Mbalu River.

### Other biting flies

It was unsurprising that biting flies other than blackflies were recovered from traps since blue and black target traps are commonly used for the collection of diurnally active haematophagous Diptera, including the genera collected during this study [63]. Given that only blood-feeding sexes of each species were recovered implies that EWTs are attractive to host-seeking flies [77].

### Consumables

Ideally, the same adhesive would have been used to coat EWTs in both Uganda and Tanzania, but this was not possible due to manufacturing problems. Both Tangle-Trap and Temmen-Insektenleim are clear, odourless adhesives commonly used to trap insects [78, 79]. They do not oxidise to form a surface film and remained sticky throughout the trapping experiments. Adhesives with these physical properties are known to be effective for collecting tsetse and other Diptera [80, 81]. Whereas the use of different products might have had an effect on the relative blackfly catch in each country, it is unlikely that this could sufficiently explain the differences in trap efficacy observed.

Differences in locally-sourced products such as sugar, yeast and container-size almost certainly affected rates of CO_2_ production in each country. Temperatures to which sugar-yeast mixtures were exposed are also likely to have had an impact. Concerns about the impact of prolonged exposure to high temperatures on CO_2_ production were addressed by conducting semi-field experiments at Gulu University (Gulu, northern Uganda) in September 2016 (S2 Fig). Experiments were conducted for four days in mean daily (07:00–18:00) temperatures of up to 36.8°C (min. 20.2°C, max. 46.0°C). Results showed that mean daily CO_2_ production did not drop below 173.79mL/min when using sugar-yeast mixtures as previously described. It is therefore also unlikely that differences in trap efficacy observed between countries were caused by effects of high temperatures on CO_2_ production. Further field-based research into the effects of consumables and environmental variables on CO_2_ production and trap efficacy is needed.

### Trap function and limitations

The choice of trap materials and their interactions with the environment affected trap performance and ease of use. The matt black emulsion initially used to paint stripes on the blue tarpaulin screen frequently peeled when removing overnight covers, although this problem was easily overcome by replacing the paint with black tarpaulin during trap construction. The adhesives used were costly if imported and affected specimen quality. It was necessary to apply a drop of white spirit to partially dissolve the glue before removing a specimen as previously recommended by Toé *et al*. (2014) [11]. This improved specimen quality, although specimen removal was consequently laborious if catch numbers exceeded 500 blackflies a day, and only a single person was working to remove them. Rodriguez-Pérez *et al*. (2013) previously stated that a single person can easily maintain five traps, and this is true providing that catch numbers are relatively low [7]. The prolonged presence of an individual at a trap also served to attract even greater numbers of blackflies. Specimen desiccation was a problem in Tanzania where blackflies were removed from traps twice daily, but was less so in Uganda where specimens were removed three times daily. It was also necessary to frequently clean traps and reapply adhesives following rainfall, which often left soil and detritus covering the base of EWTs. This was particularly important in Uganda where blackflies were mostly found on the lower third of traps.

Trap placement was particularly important to the success of collections with significant site-to-site variation in blackfly activity frequently encountered. Although no attempts were made to standardise trap placement, sites with partial shade and some direct sunlight appeared to collect most flies. Traps performed poorly in sites that were too exposed, while those placed in heavily shaded areas often caught the fewest flies.

### Conclusion

Esperanza Window Trap collections of *S*. *damnosum* s.l. in Uganda were very encouraging, with pairs of traps baited with yeast-produced CO_2_ and worn socks proving to be as efficacious as HLCs. However, successes of the Ugandan collections were not replicated in Tanzania where HLCs clearly and consistently outperformed EWTs of both colour schemes. Behavioural responses of *S*. *damnosum* s.l. to EWTs appeared to differ between study countries and this was highlighted by differences in the distribution of blackflies on traps. Responses of *S*. *damnosum* s.l. to visual and olfactory stimuli should be investigated further in East Africa given the diversity of sibling species present. Further research should also investigate whether EWTs sample the same sibling species as HLCs in areas such as Mahenge where anthropophilic and zoophilic *S*. *damnosum* s.l. occur sympatrically [35]. Since several non-anthropophilic *Simulium* species were collected on traps, it seems reasonable to assume that non-anthropophilic *S*. *damnosum* s.l. could also be present. The relatively poor performance of EWTs for the collection of anthropophilic *S*. *bovis* should raise awareness of potential limitations of EWTs for the collection of anthropophilic blackflies in areas where species other than *S*. *damnosum* s.l. transmit *O*. *volvulus*.

Current EWT designs have shown promise for the collection of *S*. *damnosum* s.l. in Burkina Faso and northern Uganda [11]. Further research and development should be encouraged to improve understanding of behavioural responses of blackflies to traps and their attractants in order to develop them as a tool for onchocerciasis surveillance in sub-Saharan Africa.

## Supporting information

S1 Fig Laboratory production of CO_2_. (PDF)

S2 Fig Semi-field production of CO_2_. (PDF)

S1 Table Trap data. (XLSX)
