# Supplementary material for: Esperanza Window Traps for the collection of anthropophilic blackflies (Diptera: Simuliidae) in Uganda and Tanzania
Source: PLoS Negl Trop Dis. 2017 Jun 19;11(6):e0005688. doi: 10.1371/journal.pntd.0005688 (PMC5491316; doi:10.1371/journal.pntd.0005688)
Supplement: S1 Fig — (PDF) [file pntd.0005688.s001.pdf]

S1 Fig. Laboratory production of CO_2.

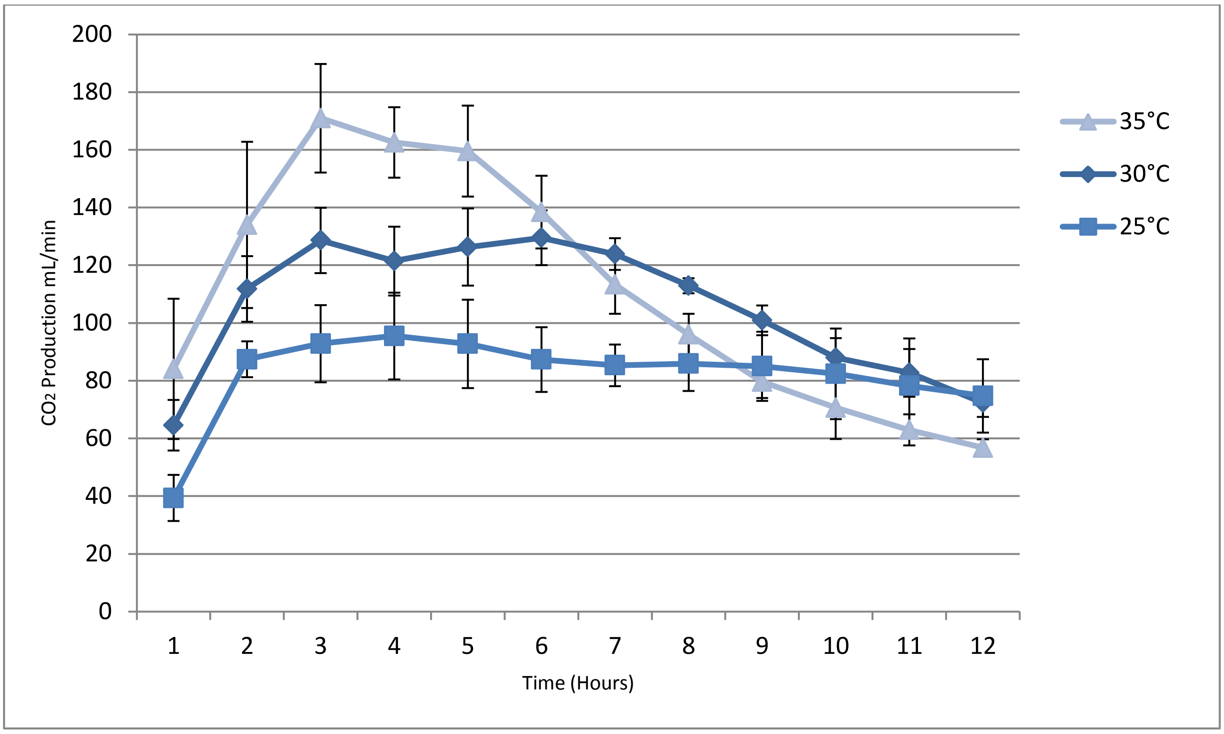

Mean values and 95% CIs of CO_2 (mL/min) produced by mixing 500g white sugar (Delhaize 365 Fine granulated sugar, Delhaize, Belgium), 50g baker's yeast (Saf-instant Red, Lesaffre, France), and 2.5L water, in a 10L container. Mixtures were incubated at 25°C, 30°C and 35°C. Measurements were made hourly for 12 hours and experiments were repeated four times at each temperature. Experiments were carried out at the Institute of Tropical Medicine, Antwerp, Belgium.
