# Supplementary material for: Esperanza Window Traps for the collection of anthropophilic blackflies (Diptera: Simuliidae) in Uganda and Tanzania
Source: PLoS Negl Trop Dis. 2017 Jun 19;11(6):e0005688. doi: 10.1371/journal.pntd.0005688 (PMC5491316; doi:10.1371/journal.pntd.0005688)
Supplement: S2 Fig — (PDF) [file pntd.0005688.s002.pdf]

S2 Fig. Semi-field production of CO_2.

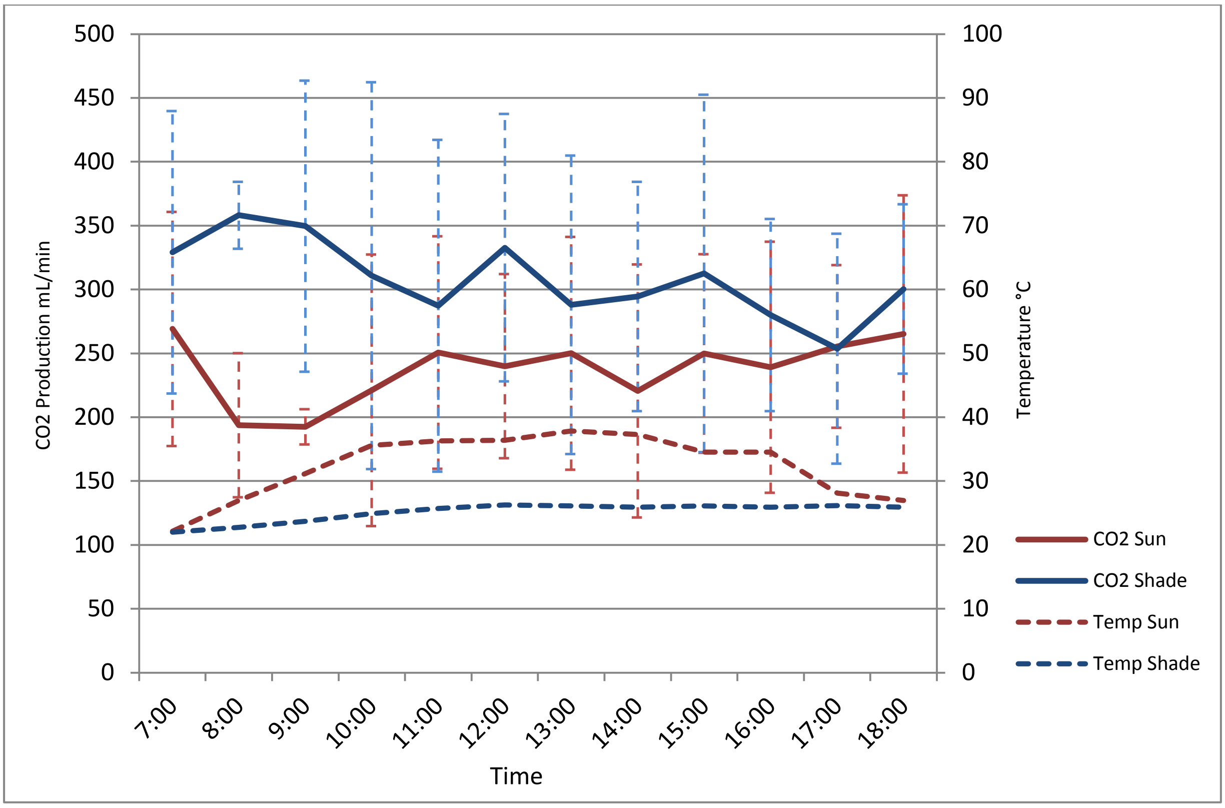

Mean values and 95% CIs of CO_2 (mL/min) produced by mixing 500g brown sugar (locally purchased, Gulu market, Uganda), 50g baker's yeast (Saf-instant Red, Lesaffre, France), and 2.5L water, in 10L containers placed in either in the sun or shade at Gulu University, Uganda. Measurements were made hourly for 11 hours (07:00 – 18:00) and were repeated for four consecutive days.
